# Luminescent upconversion nanoparticles evaluating temperature-induced stress experienced by aquatic organisms owing to environmental variations

**DOI:** 10.1016/j.isci.2022.104568

**Published:** 2022-06-09

**Authors:** Alexey Popov, Maxim Timofeyev, Alexander Bykov, Igor Meglinski

**Affiliations:** 1 VTT Technical Research Centre of Finland, 90590 Oulu, Finland; 2 Institute of Biology, Irkutsk State University, Irkutsk 664003, Russia; 3 Baikal Research Centre, Irkutsk 664003, Russia; 4 Optoelectronics and Measurement Techniques, ITEE, University of Oulu, Oulu 90579, Finland; 5 Institute of Engineering Physics for Biomedicine, National Research Nuclear University (MEPhI), Moscow 115409, Russia; 6 Interdisciplinary Laboratory of Biophotonics, Tomsk State University, Tomsk 634050, Russia; 7 Histology, Cytology and Embryology Department, I.M. Sechenov First Moscow State Medical University, Moscow 119991, Russia; 8 REC «Fundamental and Applied Photonics, Nanophotonics», Immanuel Kant Baltic Federal University, Kaliningrad 236041, Russia; 9 College of Engineering and Physical Sciences, Aston University, Birmingham B4 7ET, UK

**Keywords:** Environmental science, Aquatic science, Zoology, Physiology

## Abstract

Growing anthropogenic activities are significantly influencing the environment and especially aquatic ecosystems. Therefore, there is an increasing demand to develop techniques for monitoring and assessing freshwater habitat changes so that interventions can prevent irrevocable damage. We explore an approach for screening the temperature-induced stress experienced by aquatic organisms owing to environmental variations. Luminescent spectra of upconversion [Y2O3: Yb, Er] particles embedded within *Caridina multidentata* shrimps are measured, while ambient temperature gradient is inducing stress conditions. The inverse linear dependence of the logarithmic ratio of the luminescence intensity provides an effective means for temperature evaluation inside aquatic species *in vivo*. The measured luminescence shows high photostability on the background of the complete absence of biotissues’ autofluorescence, as well as no obscuration of the luminescence signal from upconversion particles. Current approach of hybrid sensing has a great potential for monitoring variations in aquatic ecosystems driven by climate changes and pollution.

## Highlights

• Luminescence spectra induced by upconversion particles are embedded into aquatic animals
• Real-time quantitative assessment of temperature inside aquatic species *in vivo*
• Evaluation of stress handled by water organisms owing to environmental variations
• Hybrid sensing approach for monitoring environmental variations driven by climate change

## Graphical abstract

**Figure:**
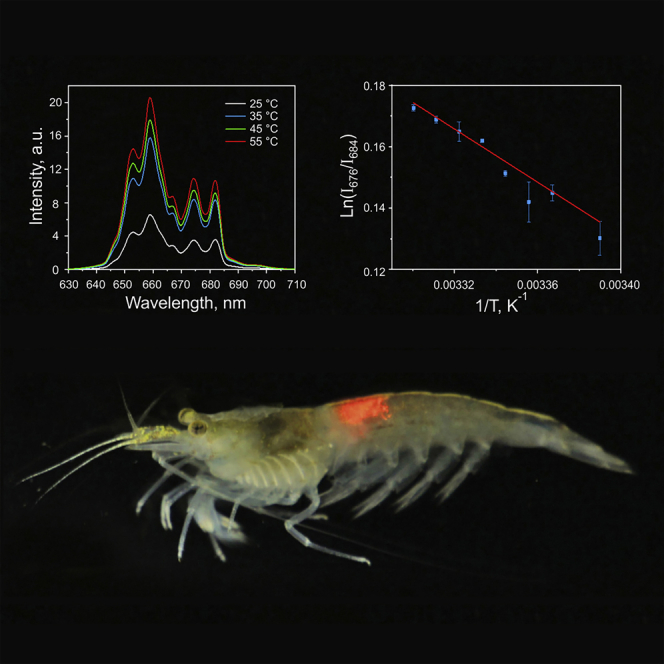

## Introduction

The global, rapidly accelerating challenges of environmental pollution and climate change are driving a growing interest in localized biosphere changes. Humanity is currently facing unprecedented environmental changes on a global scale. Meteorological studies carried out in regions all over the world have consistently shown that the climate is in the process of changing. The average global temperature is one of the most cited indicators for this. Since the beginning of the 20th century, this temperature metric has increased by 0.74 ± 0.18°C (IPCC, 2007). There has been a clear, long-term global warming trend, although not every individual year shows a temperature increase over the previous year. Recent models predict an increase in the average global temperature of up to 5°C by the end of the 21^st^ century, with increased temperature fluctuations and extremes (IPCC, 2007; 2013). The warming trend, visible from all independent methods of the calculation of the global temperature change, is also accompanied by other observations. There have been, for example, extremely hot summers, more frequent hurricanes, melting of mountain glaciers on all continents, reduction of the snow cover thickness, earlier onset of the blossoming of plants in spring, a shorter ice season on lakes and rivers, accumulated heat in the ocean, shrinking of the Arctic ice and rising sea levels, to name a few. In short, climate change is not just global warming. Different local environments can be affected in radically different ways, characterized by exceptionally complex feedback loops of cause and effect.

Simultaneously, the biosphere is currently heavily affected by anthropogenic pollution. The number of different man-made chemicals now used in industry and agriculture exceeds 100,000, with 10,000–30,000 being of environmental concern (Hartung and Rovida, 2009). The combination of climate change effects and further exacerbation by anthropogenic activities will greatly impact both the biosphere and mankind (Delworth and Zeng, 2014; Hoegh-Guldberg and Bruno, 2010; Lobell et al., 2011; IPCC, 2007, 2013). The variety of possible reactions of ecosystems in response to global climate change makes predictions of the possible consequences extremely complex. According to a report from the World Wildlife Fund and the Zoological Society of London (World Wildlife Fund, 2016), the total number of marine mammals, birds, fish, and reptiles has decreased by 58% between 1970 and 2012. Changes have been especially prevalent in aquatic ecosystems (Kashulin et al., 2012; IPCC, 2007). For example, the extant populations of mackerel and tuna in the oceans have decreased by 74% in just the last 45 years.

Freshwater ecosystems are particularly vulnerable to the combination of both temperature changes and chemical pollution. Industrial and urban wastewater, as well as agricultural runoff, often ends up in rivers and lakes creating a continuous inflow of chemicals into aquatic ecosystems. Alterations in the ambient temperature entail the intensification of various processes causing stress and further destruction of aquatic ecosystem populations. Elevated temperature in combination with chemical stressors results in increased overall stress for aquatic organisms (Lannig et al., 2008). However, these environmental changes can be advantageous for species with a superior ability to cope with these stress conditions. They become able to outcompete species that are well-adapted to specific conditions but with less stress tolerance. Owing to the introduction and spreading of non-native species, there is a trend toward the global biosphere becoming less diverse. Climate and chemosphere change favor global “generalists” that outcompete indigenous and endemic fauna. Mass occurrences of invasive species are often observed in heavily anthropogenically affected ecosystems (Grabowski et al., 2007; Van Den Brink et al., 1991). Global biosphere changes can cause significant losses in ecological and economic resources around the world, resulting in heavy long-term costs to society (Mäler, 2000; Scheffer et al., 2001).

The use of optical sensors for rapid assessment, and both quantitative and qualitative analysis of living organisms *in vivo*, is an extremely promising research area for biomedical and environmental screening (D’Orazio, 2011). Over the past few decades, a variety of sensors and sensing devices, utilizing the physicochemical relationships between various compounds, have been developed in close collaboration between biologists and engineers (Gunatilaka and Diehl, 2001; Kovalska et al., 2019; Li et al., 2015; Luchansky and Bailey, 2012; Mumby et al., 2004). Many of these sensors monitor changes in the electrical properties of the detector during and after exposure to a substance (Kovalska et al., 2019). Sensors based on the optical response of a fluorescent dye which reacts to a specific substance, or to physiological changes in the medium, have also been developed (Moczko et al., 2009; Saxl et al., 2009). The use of such optical diagnostic approaches is considered particularly promising, as these techniques allow direct non-invasive screening of living organisms (Grigoriev and Miller, 2009; Ruckh and Clark, 2014).

Environmental monitoring of water basins and rapid diagnosis of physiological states of living organisms *in vivo* are areas of great promise for the application of optical sensors. Current techniques for the determination and analysis of physiological parameters, for small organisms, in particular, do not allow measurements to be recorded without the destruction of the organisms (Bownik et al., 2015). Because of this distinct disadvantage, in ecological and ecophysiological studies exploring the abilities of aquatic organisms to adapt to negative environmental factors, or when testing water quality, different individuals at each exposure point must be used. This significantly decreases the precision of measurements owing to interindividual variations and significantly increases the time and cost of the studies.

Many fluorescent sensors allowing a comprehensive assessment of the physicochemical characteristics of physiological fluids are currently commercially available. However, these existing sensors are typically toxic. Therefore, they cannot be applied directly to the diagnosis of living organisms. Other critical issues that must still be addressed are the biodegradability of the sensors, and difficulties associated with obtaining a suitably strong signal from the substance when it is homogeneously distributed in the medium. To avoid these problems, the encapsulation of fluorescent sensors with a semi-permeable membrane has been proposed (Sadovoy et al., 2012). This approach allows one to maintain the sensor’s specificity to particular compounds in the medium, while simultaneously preventing direct contact of the fluorescent sensors with the medium, thus avoiding the problem of toxicity. This approach also increases the sensor lifetime and allows for localizing it in a specific area, facilitating signal detection. Overall, the encapsulation of optical sensors has the potential to provide next-generation diagnostics and measurement of the physiological parameters of living organisms, both remotely and in real-time (Borvinskaya et al., 2016, 2017, 2018a, 2018b; Gurkov et al., 2016, 2017).

Monitoring temperature changes can act as a proxy measurement for environmental stress factors affecting living organisms. Factors such as changing metabolic rates are reflected in temperature changes, as well as the direct influence of the environmental temperature (Shadwick et al., 2013; Rajagopal et al., 2019; Wang et al., 2021). A precise and accurate *in vivo* temperature measurement technique can, therefore, play an important role in monitoring organisms’ health. Fluorescent nanoparticles have previously been applied to measure thermal variations in cells (Axenov-Gribanov et al., 2011; Yang et al., 2011). For example, temperature variations within biological tissues can be observed *in vivo* in real-time by monitoring the fluorescence spectra of temperature-sensitive ZnCdS semiconductor nanoparticles embedded into muscles or skin (Martinez Maestro et al., 2010). Different types of nanomaterials, such as carbon nanotubes, silver nanospheres, gold nanoparticles, and quantum dots (CdSe, CdTe, ZnCdS), for which the luminescence properties depend on the ambient temperature, have also been considered (Martinez Maestro et al., 2011; Volkova, et al., 2015). A further area of interest is phosphorescent nanoparticles. The majority of phosphors used today as nano-thermometers emit luminescence by the down-conversion mechanism and require high power excitation (Huang et al., 2006). In contrast, anti-Stokes luminescent nanoparticles absorb multiple photons in the near-infrared (NIR) spectral range followed by emission in the visible/NIR range, employing an upconversion mechanism (Chatterjee and Yong, 2008; van Rhoon and Wust, 2005). There is strong potential for the use of up-conversion nano-particles (UCP) in the detection of temperature variations in biological tissues in the visible spectral range. Interest in their use, as a replacement for quantum dots, is on the rise owing to their high photochemical stability, low toxicity, and small sizes (Guller et al., 2015; Popov et al., 2015). Importantly, in the luminescence spectral region of UCP there is a marked absence of autofluorescence from biotissues, thus efficiently eliminating the problem of background signals from the biological tissue (Martinez Maestro et al., 2010).

## Results and discussion

**Figure 1:**
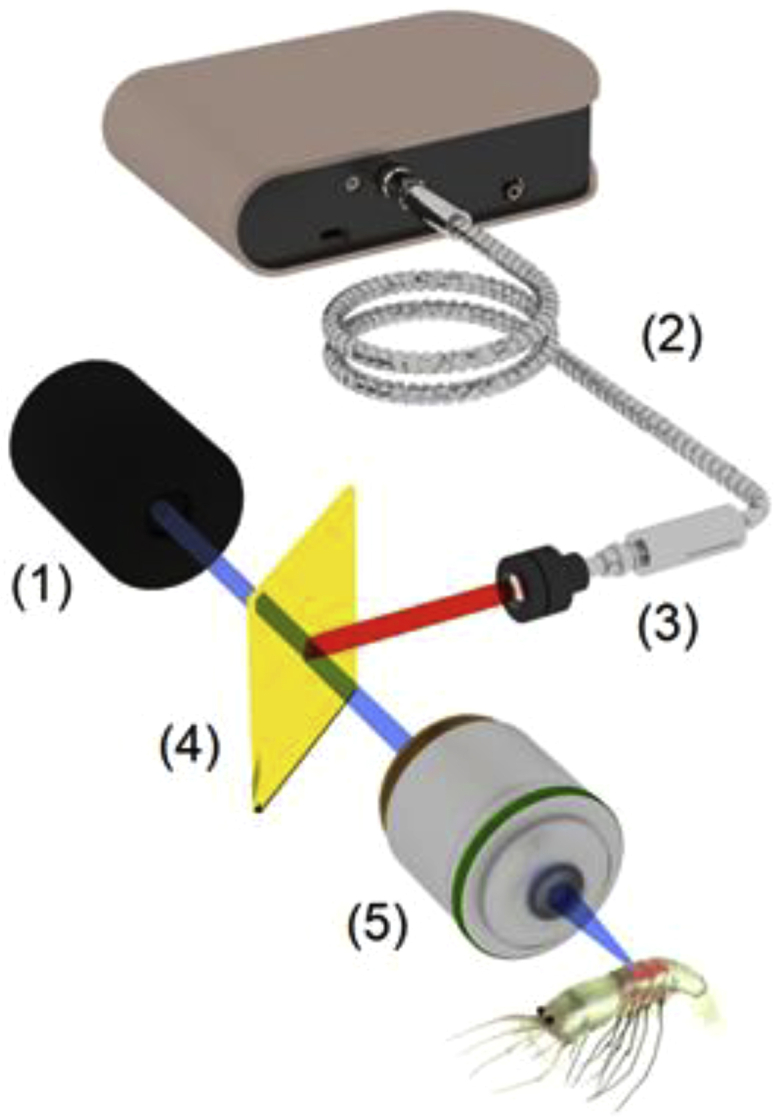
Experimental protocol/measurements Schematic overview of the experimental protocol/measurements, utilizing standard laser diode as a light source (1) and portable spectrometer (2), equipped with the detecting optical fiber (3), dichroic mirror (4) and microscopy objective (5) to deliver light to/from the object of interest.

Herein, the development of an approach for non-invasive monitoring of the stress conditions in water organisms is presented. The application of [Y_2_O_3_: Yb, Er] UCP for real-time quantitative assessment of internal temperature in small aquatic species is detailed, focusing on *Caridina multidentata* shrimp (CMS). Commercially available hydrophilic anti-Stokes phosphors UCP with an average particle size (∼ 1.4 μm) and size deviation (∼0.4 μm), maximum excitation at 950 and 975 nm, and maximum emission at 661, 676, and 683 nm, respectively, were used to sense the local temperature within small aquatic species. To observe the luminescent signal from the anti-Stokes phosphors placed in the biological tissue, we used an experimental setup schematically shown in Figure 1.

**Figure 2:**
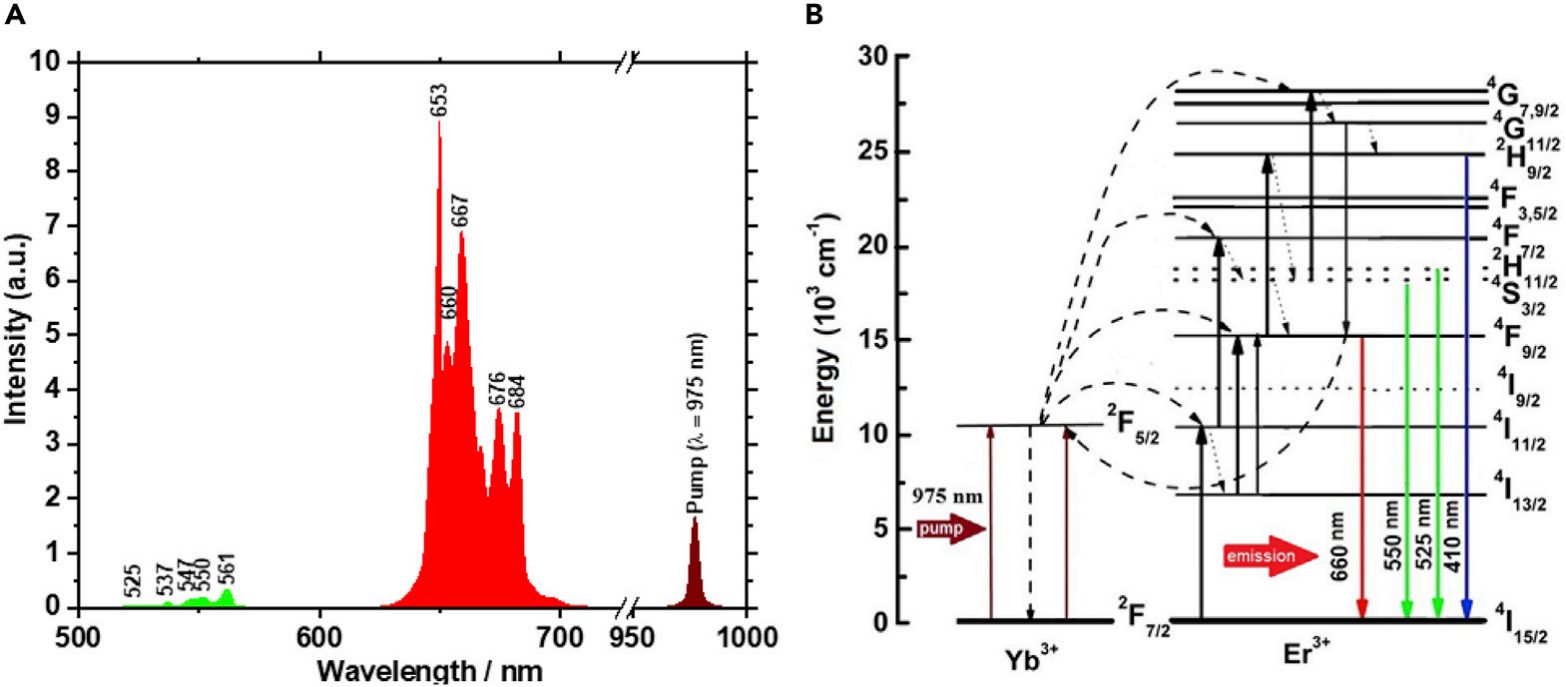
Luminescence spectrum of UCP and energy levels The luminescence spectra of UCP at 25°C with an excitation wavelength of 975 nm (A). Schematic representation of the energy levels and energy transfer in the [Y_2_O_3_: Yb^3+^, Er^3+^] crystal (B).

The luminescence spectrum of the UCP at 25°C is shown in Figure 2A. For the quantitative analysis of environmental influences on the particle luminescence, the intensity peaks at the wavelengths of λ_1_ = 676 nm and λ_2_ = 684 nm were chosen. These wavelengths were selected owing to having the highest sensitivity of their ratio to changes in the ambient temperature during the experiments. The upconversion process is illustrated by the energy diagram (Figure 2B). Luminescence in the red region of the spectrum occurs when Er^3+^ transitions from the ^4^F_9/2_ state to the ground state. The relative intensity of the UCP luminescence at a given wavelength is governed by the Boltzmann distribution (Vetrone et al., 2010):

(Equation 1) $$\frac{I\left(\lambda_{1}\right)}{I\left(\lambda_{2}\right)}=C\;\mathrm{ex}p^{-\frac{\text{Δ}E}{kT}},$$

where: *I*(*λ*_*1*_)/*I*(*λ*_*2*_) is the ratio of the luminescence intensities at the wavelengths *λ*_*1*_ = 676 nm and *λ*_*2*_ = 684 nm; *C* is a constant depending on the degeneracy, spontaneous emission rate, and photon energies of the emitting ion states in the UCP; *ΔE* is the energy gap separating the two excited states; *k* is the Boltzmann constant; and *T* is the absolute temperature.

Using this relationship, a relative temperature change can be calculated from the relative luminescence intensities as:

(Equation 2) $$F=ln\left(\frac{I\left(\lambda_{1}\right)}{I\left(\lambda_{2}\right)}\right)=lnC-\frac{\text{Δ}E}{kT},$$

We define the natural logarithm of the intensity ratio between the wavelengths λ_1_ = 676 nm and λ_2_ = 684 nm as the F-factor. The F-factor is an experimentally measurable quantity, which can then be used for the quantitative assessment of temperature changes.

Water-suspended UCP were tested to establish the temperature dependence of the luminescence.

**Figure 3:**
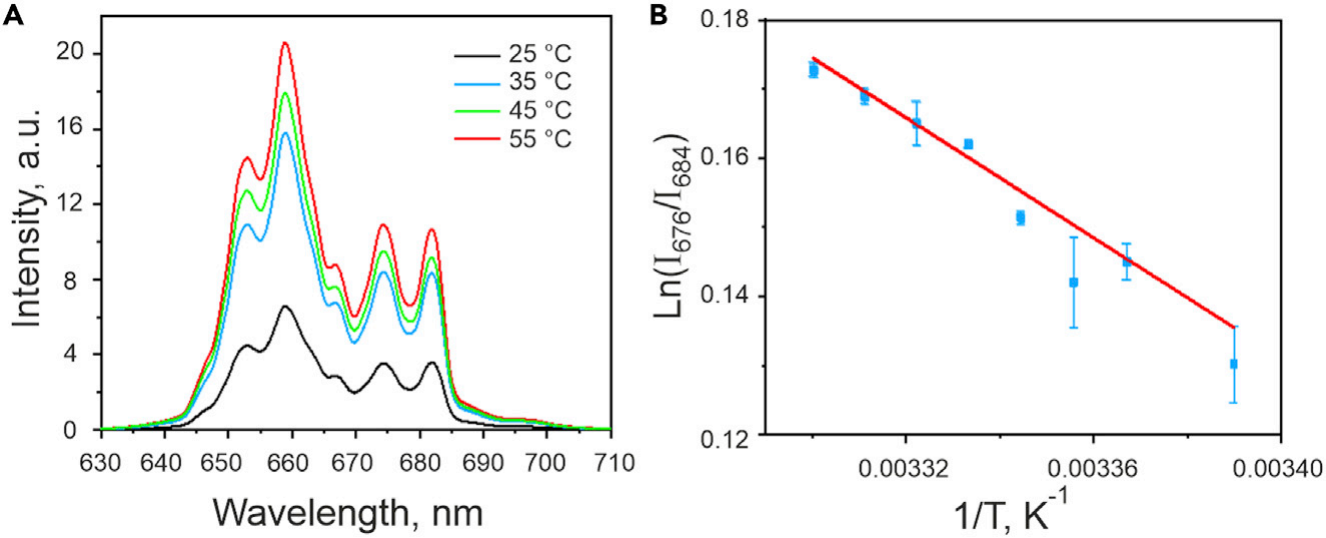
Luminescence spectra of water-suspended UCP and F-factor Luminescence spectra of water-suspended UCP in the red spectral range at different temperatures for an excitation wavelength of 975 nm (A). Temperature dependence of the F-factor for UCP suspended in pure water, with an excitation wavelength of 975 nm (B). The error bars are standard deviation values resulting from the averaging.

Luminescence spectra of the UCP in the red spectral range for 25-55°C temperatures are shown in Figure 3A. The calculated F-factor is inversely proportional to the environmental temperature increase (Figure 3B), allowing the quantitative assessment of temperature. The symbols correspond to mean values averaged over five measurements. The error bars represent standard deviations.

**Figure 4:**
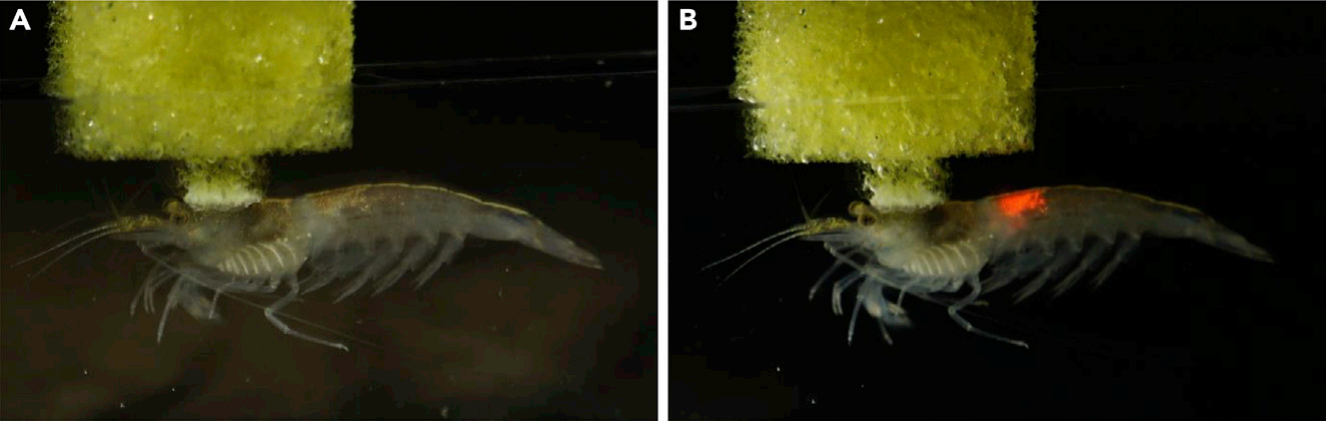
Images of CMS *in vivo* Images of CMS *in vivo:* before (A) and after (B) illumination with a 975 *nm* laser.

Figure 4 shows images of an individual CMS illuminated with a laser before (Figure 4A) and after the injection of UCP into the animal (Figure 4B). Upon illumination with 975-nm laser light, the luminescence of the UCP appears in its abdominal and head-thorax areas (see Figure 4B).

**Figure 5:**
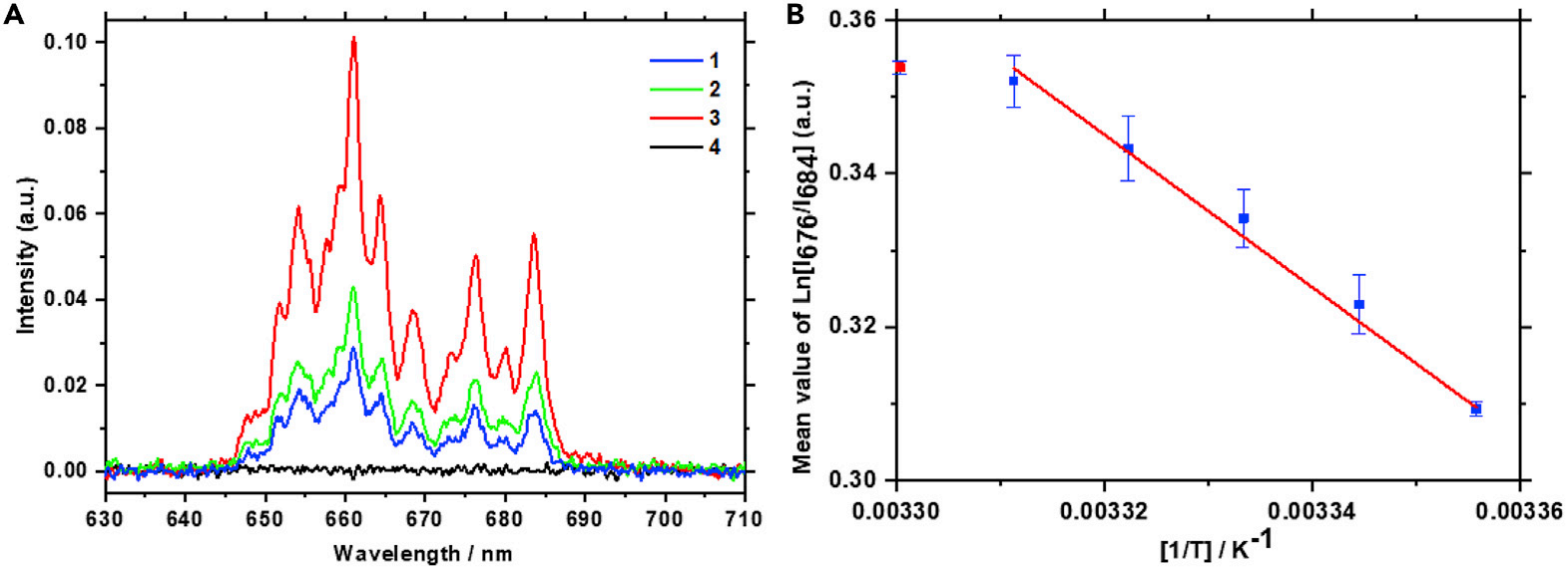
Luminescence spectra of the UPC inside CMS and F-factor (A) Typical luminescence spectra of the UPC inside CMS at 21°C (1, blue) and 45°C (2, green). Luminescence bands of the water-suspended UPC (3, red) at 22°C, and autofluorescence of the shrimp at 21 °C (4, black). (B) – Temperature dependence of the F-factor for UPC injected into CMS, with an excitation wavelength of 975 nm. The error bars are standard deviation values resulting from the averaging.

Experiments with water-suspended UCP were used as a reference for data obtained during measurements with shrimps. Luminescence spectra of the UCP in the red spectral range inside CMS *in vivo* for different temperatures are shown in Figure 5A (1, blue and 2, green). It is clearly seen that during excitation by the laser light (λ = 975 nm), no auto-fluorescence of the shrimp in the luminescence range of UCP is present (4, black). Thus, there is no obscuration of the luminescence signal from the UCP.

Similar to Figure 3B, a linear dependence on inverse temperature of the UCP luminescence is observed for particles injected into CMS (Figure 5B). The change in the anthropogenic chemical composition of the environment, between pure water and physiological medium inside the shrimp, affects the *C* constant in (Equation 2), resulting in the shift of the data points’ position (compare the numbers on the vertical axes in Figures 3B and 5B).

It should be pointed out that the error bars in Figure 5B are not the same as SD in Figure 3B, as the experiments are conducted in different media. F-factor of the temperature dependence of particles injected into the animals does not really have a more even distribution of errors (Figure 5B) than particles in water only (Figure 3B): some of the error bars are small but some are large in Figure 3B, while in Figure 5B the error bars are more homogeneous. This is explained by the fact that the shrimp as a medium (Figure 5B) is far more complex than water (Figure 3B).

The most prominent advantages of UCP, in comparison with commonly used dye-based fluorescent analogs, are their high photostability and the total absence of biotissue auto-fluorescence, a significant factor interfering with the meaningful optical signal (Jacewicz et al., 2017). The advantages of UCP ensure their higher signal-to-noise ratio in comparison with the above-mentioned fluorophores, and a strong potential for application in different areas of physiological measurements. In particular, UCP can be used for direct *in vivo* temperature monitoring in experimental setups with high-temperature variability within the aquatic media or for the evaluation of animals’ metabolic rates. The latter is critical in studying the effects of global warming and environmental pollution (Ern et al., 2015). Thus, this environmental-based oriented research area has a great potential for monitoring anthropogenic environmental change and can provide a deeper ultimate understanding of organisms’ physiology and toxicology, as well as the mechanisms of their adaptation, through accurate and precise environmental analysis and diagnosis.

### Conclusions

We introduce an approach for the non-invasive screening of stress felt by water organisms owing to environmental variations driven by climate change. The capability for real-time *in vivo* quantitative assessment of temperature inside aquatic species, such as *C. multidentata* shrimp, is demonstrated. The experimental approach is based on measurements of the luminescence of UCP embedded into the aquatic animal. An inverse linear dependence of the logarithmic intensity ratio of two “red” lines within the luminescence spectrum toward the temperature increase is observed. These results demonstrate the capability of the use of the UCP embedded in living aquatic organisms as an implantable sensor for monitoring stress handled by water organisms owing to environmental variations. The presented technique is applicable to the study of many other aquatic biosystems, from other invertebrates to fish. This environmental-based-oriented research area has great potential for monitoring anthropogenic environmental change and can provide a deeper ultimate understanding of organisms’ physiology and toxicology, as well as the mechanisms of their adaptation, through accurate and precise environmental analysis and diagnosis.

### Limitations of the study

Both climate change and anthropogenic chemicals in the environment greatly impact the biosphere and mankind. Freshwater ecosystems are typically affected by both temperature changes and pollution associated with anthropogenic activities. Thus, industrial and urban wastewater, as well as agricultural runoff, often end up in rivers and lakes, which create a continuous inflow of contamination into aquatic ecosystems. Elevated temperature in combination with pollution stressors results in increased overall stress for the water species. The differentiation of contaminations and particular stressors identification with UCP is not imaginable at the current stage. The microinjection of UCP into the body of species becomes an impediment for a wide use such hybrid sensing approach in a routine day-to-day environmental monitoring. Alternatively, owing to the lower abundance of stress markers while using UCP as additives during feeding the species, the detection of luminescence excitation can’t be achieved with high resolution and/or requires additional optimization.

## STAR★Methods

### Key resources table

**Table:** 

REAGENT or RESOURCE	SOURCE	IDENTIFIER
**Biological samples**		

*Caridina multidentata* shrimp	AKVAARIOKESKUS KY	Cat# HCR8973

**Chemicals, peptides, and recombinant proteins**		

Upconversion [Y_2_O_3_: Yb, Er] particles	Phosphor Technology, UK	PTIR660-UF UCPs

**Other**		

laser diode	Thorlabs, USA	PL980P330J
color bandpass filter	Thorlabs, USA	FB600-10
spectrometer	Thorlabs, USA	CCS200

### Resource availability

#### Lead contact

Further information and requests for resources and reagents should be directed to and will be fulfilled by the lead contact Igor Meglinski (i.meglinski@aston.ac.uk).

#### Materials availability

This study did not generate new unique reagents.

### Experimental model and subject details

In this study, the research object is *Caridina multidentata* shrimp (CMS), and no experimental models.

### Method details

#### Method

To sense the local temperature of the biological tissue, we used commercially available hydrophilic PTIR660-UF UCPs (Phosphor Technology, UK). To observe the luminescent signal from the anti-Stokes phosphors UCP placed in the water species, we used an experimental setup schematically shown in Figure 1. The luminescence from UCP was excited by a laser diode PL980P330J (λ = 975 nm, Thorlabs, USA). The luminescence spectra were measured at a distance of 25 mm from the surface of the biological sample through an optical fibre sensor equipped with a colour bandpass filter FB600-10 (Thorlabs, USA) and a 20× objective. Photoluminescence signals were recorded by a CCS200 spectrometer (Thorlabs, USA) with an acquisition time of 200 ms. Laser irradiation for each measurement lasted for 5 seconds. A laser output power of 25 mW, just above the lasing threshold, was used to minimize the laser-induced temperature effects.

#### Materials

The experiments were conducted *in vivo* and in accordance with the principles of the Basel Declaration. Four adult individual CMS decapods were used as biological samples. The whole abdominal cavity of the shrimps is filled with hemolymph pumped throughout the body by means of the heart. UCP, suspended in a phosphate buffered saline to form a slurry (10 μL, 90% solid content), were injected into the abdominal region of the decapods using an insulin syringe immediately prior to the experiment. The shrimps were held in a fixed position during measurements by means of a degradable and removable glue. The temperature of the cuvette with the shrimp was increased from 25°C to 30°C with a 1°C step using a Peltier element equipped with a temperature control sensor. The whole experiment took place over about 20 minutes for each aquatic animal. Afterwards, the shrimps were kept in an oxygenated aquarium and were fed normally. They survived for at least two weeks after the imaging experiments, demonstrating the low toxicity of the used UCP.

### Quantification and statistical analysis

Standard error of the mean is shown on figures unless otherwise noted. The study does not include any specific quantification and statistical analysis.

## Data Availability

The published article include all data generated and analysed during this study. This paper does not report original code and a supplemental information. Any additional information required to reanalyse the data reported in this paper is available from the Lead contact upon request.
